# Pegylated Interferon-*α* Modulates Liver Concentrations of Activin-A and Its Related Proteins in Normal Wistar Rat

**DOI:** 10.1155/2015/414207

**Published:** 2015-07-06

**Authors:** Bassem Refaat, Adel Galal El-Shemi, Ahmed Mohamed Ashshi, Elaf Wael Mahamid, Noha Mohammed Al-Qadi

**Affiliations:** ^1^Laboratory Medicine Department, Faculty of Applied Medical Sciences, Umm Al-Qura University, P.O. Box 7607, Al Abdeyah, Makkah, Saudi Arabia; ^2^Department of Pharmacology, Faculty of Medicine, Assiut University, Assiut 6515, Egypt

## Abstract

*Aims*. To measure the expression of activin *β*A-subunit, activin IIA and IIB receptors, Smad4, Smad7, and follistatin in the liver and the liver and serum concentrations of mature activin-A and follistatin in normal rat following treatment with pegylated interferon-*α* (Peg-INF-*α*) and ribavirin (RBV). *Materials and Methods*. 40 male Wistar rats were divided equally into 4 groups: “control,” “Peg-only” receiving 4 injections of Peg-INF-*α* (6 *µ*g/rat/week), “RBV-only” receiving ribavirin (4 mg/rat/day) orally, and “Peg & RBV” group receiving both drugs. The expression of candidate molecules in liver was measured by immunohistochemistry and quantitative PCR. The concentrations of mature proteins in serum and liver homogenate samples were measured using ELISA. *Results*. Peg-INF-*α*  ± RBV altered the expression of all candidate molecules in the liver at the gene and protein levels (*P* < 0.05) and decreased activin-A and increased follistatin in serum and liver homogenates compared with the other groups (*P* < 0.05). There were also significant correlations between serum and liver activin-A and follistatin. *Conclusion*. Peg-INF-*α* modulates the hepatic production of activin-A and follistatin, which can be detected in serum. Further studies are needed to explore the role of Peg-INF-*α* on the production of activins and follistatin by the liver and immune cells.

## 1. Introduction

Infection with hepatitis C virus (HCV) is a global health problem and it is a leading cause for the development of liver fibrosis, cirrhosis, and hepatocellular carcinoma [1]. Although new directly acting antiviral drugs (DAAs) have been developed for the treatment of chronic hepatitis C (CHC), these drugs are expensive and therefore pegylated interferon-*α*- (Peg-INF-*α*-) 2a or 2b plus a daily weight-based dose of ribavirin could still be the standard of care especially for the treatment of naïve patients with compensated liver functions and/or for those living in developing countries and for whom access to the new drugs is not definite [2–7]. In this regard, a large proportion of CHC patients lack adequate insurance limits to cover their access to DAAs [4]. Even in wealthy countries, reimbursement of DAAs is only allowed for CHC patients who are interferon unable, with advanced liver disease and/or transplanted organs due to the budget-breaking price of these oral medicines [5, 6]. Hence, one possible approach to overcome the high cost of the DAAs could be by identifying those patients who could respond to the conventional Peg-INF-*α* based therapy as a first line of treatment [2, 3, 5].

The development of hepatic complications following infection with HCV is due to the promotion of adaptive immune response by activating T helper (Th) 2 pathway [8, 9]. IFN-*α* alters the immune response in patients with CHC from Th2 to a Th1 mediated pattern, which favours the eradication of the virus [10, 11]. INF-*α* induces Th1 response through the modulation of several cytokines including IFN-*γ*, tumour necrosis factor- (TNF-) *α*, interleukins (IL), and transforming growth factor- (TGF-) *β* by the hepatocyte and immune cells [12–14].

Activins are members of the TGF-*β* family and their biological activities are tightly regulated by their binding protein follistatin [15]. Similar to any extracellular protein signals, activins execute their actions by binding to cell membrane receptors, namely, activin type I and type II receptors. Activins can bind to their individual receptor type II (IIA and IIB) when expressed alone, but they fail to bind to type I receptor in the absence of the type II receptor [15]. However, both receptor types are necessary to generate a high-affinity complex with activins, as well as for signalling. The activated activin type I receptor propagates the signal through the phosphorylation of other proteins known as Smad proteins [16, 17].

There are three functional classes of Smads: the receptor-regulated Smad (R-Smad), the comediator Smad (Co-Smad), and the inhibitory Smad (I-Smad). The R-Smads (Smad1, Smad2, Smad3, Smad5, and Smad8) are directly phosphorylated and activated by the type I receptor kinases and undergo homotrimerization and formation of heteromeric complexes with the Co-Smad, known as Smad4. The activated Smad4 then enters the nucleus and initiates transcription of specific genes by the association with other regulatory factors [16, 17]. Smad6 and Smad7, both are known as I-Smads, negatively regulate TGF-*β* signalling by competing with the R-Smads for receptor or Co-Smad interaction and by targeting the receptors for degradation [16, 17].

Activin-A and follistatin are expressed by the hepatocyte and have been described as major regulators of liver biology, liver regeneration, and liver pathology [18]. Additionally, they play an important role in the regulation of the immune system and the pathogenesis of inflammatory and fibrotic human diseases [19]. Activin-A and follistatin have been proposed as diagnostic/prognostic markers for a variety of liver diseases since pathological alterations in their serum concentrations, which correlated with the severity of the diseases, have been documented in several liver pathologies including viral hepatitis B and hepatitis C [20, 21].

We have previously reported that CHC and Peg-INF-*α* based therapy modulate the serum concentrations of activin-A and follistatin and we have postulated that Peg-INF-*α* alters the serum concentrations of these proteins by regulating their production in the liver [22, 23]. Hence, the present study was conducted to measure the effects of Peg-INF-*α* based therapy on the expression of activin *β*A-subunit, activin type IIA and IIB receptors, Smad4, Smad7, and follistatin in liver tissue collected from normal Wistar rat since it is not ethically possible to inject healthy human subjects with unnecessary drugs. Furthermore, the effects of Peg-INF-*α* based therapy on the concentrations of the mature activin-A and follistatin proteins were measured in liver homogenates and serum samples collected from the animals.

## 2. Materials and Methods

### 2.1. Drugs

Pegylated interferon-*α*-2a (Pegasys, Hoffmann-La Roche, Nutley, NJ) was used. The ready to use syringe contains 180 *μ*g/0.5 mL. Ribavirin capsules (Viracure, 6th October Pharm, Egypt) were used and each capsule contains 400 mg of ribavirin.

### 2.2. Study Design

All experimental protocols were approved by the Committee for the Care and Use of Laboratory Animals at Umm Al-Qura University and were in accordance with the EU Directive 2010/63/EU for animal experiments.

A total of 40 male Wistar rats weighing 250–300 gm were used. All animals received humane care during the study protocol and during euthanasia. The animals were divided equally into 4 groups as follows: the first group included 10 rats and they served as “control” group, the second group consisted of those that only received Peg-INF-*α* “Peg-only” group, the third received ribavirin only “RBV-only” group, and the last group consisted of rats that received both Peg-INF-*α* and ribavirin “Peg & RBV” group.

The study duration was 5 weeks. Peg-INF-*α*-2a was prepared by diluting the content of a full syringe (0.5 mL) in normal saline to prepare a final volume of 10 mL and the final concentration was 18 *μ*g/mL. Each rat in the “Peg-only” and “Peg & RBV” groups received a weekly subcutaneous injection of 0.33 mL (6 *μ*g/rat). Each rat received a total of 4 injections. One capsule of ribavirin (400 mg) was dissolved in 50 mL saline and each rat in the “Peg & RBV” and “RBV-only” groups received 0.5 mL (4 mg)/day orally using a feeding syringe for the whole length of the study similar to the highest dose of the drug recommended from human during CHC treatment (12 mg/kg [1200 mg for body weight ≥75 kg]) [24]. Following 4 injections, the rats were sacrificed in the fifth week on the same day the 5th injection would have been given. Ribavirin was continued till the day before euthanasia.

### 2.3. Types of Samples

All rats were sacrificed on the same day under diethyl ether anaesthesia (Fisher Scientific UK Ltd., Loughborough, UK) a week after the last injection and 4 mL of blood was collected in plain tube immediately after cutting the vena cava. Blood samples were centrifuged for 20 minutes and the serum was stored in −20°C for routine biochemistry and to measure serum concentrations of activin-A and follistatin.

Two specimens from the middle lobe of the liver (2 gm/each) were obtained from each animal with one piece being immediately processed for protein extraction using 6 mL of RIPA lysis buffer containing protease inhibitors (Santa-Cruz Biotechnology Inc., Burlingame, CA) and electrical homogeniser. All samples were centrifuged at 14000 rpm for 30 minutes and small aliquots (0.5 mL) of the resultant supernatant were placed in Eppendorf tubes and stored in −20°C till processed to measure the levels of candidate proteins in liver using ELISA.

The other liver specimen was divided equally with one piece that was immediately fixed in 10% buffered formalin for 24 h at room temperature, dehydrated with a series of different ethanol concentrations, and embedded in paraffin for immunohistochemistry. The remaining portion was immersed in 5 mL of RNA*later* (Ambion, Thermo Fisher Scientific, USA) and stored in −80°C to preserve their RNA stability for quantitative RT-PCR.

### 2.4. Immunohistochemistry

Polyclonal goat IgG antibodies to detect activin *β*A-subunit (C-18), activin type IIA (N-17) and IIB (N-16) receptors, Smad4 (C-20), Smad7 (P-20), and follistatin (K-19) were obtained from Santa-Cruz Biotechnology Inc. (Burlingame, CA).

An avidin-biotin horseradish peroxidase technique was used to localize the proteins of interest. Briefly, paraffin embedded sections of 5 *μ*m thickness were dewaxed in xylene, dehydrated in alcohol, and treated with 2% (vol/vol) hydrogen peroxide for 20 minutes in methanol to block endogenous peroxidase. All sections were pretreated in an 850-watt domestic microwave oven in 0.01 M citrate buffer for 3 minutes. The sections were incubated for 30 minutes with normal donkey serum. The sections were then incubated with the primary antibodies (the antibody concentration was 1 : 100 for all used antibodies) overnight at 4°C.

The following day the sections were washed with 20 mM PBS (pH 7.3) and then incubated with 1 : 200 biotinylated anti-goat secondary antibody for 30 minutes. After a further wash step, the sections were incubated with the avidin-biotin peroxidase complex ABC system (Santa-Cruz Biotechnology Inc., Burlingame, CA) for 30 minutes and then subsequently with 3,3′-diaminobenzidine (Santa-Cruz Biotechnology Inc., Burlingame, CA) for 10 minutes. Sections were washed in tap water, counter-stained with Gill's haematoxylin, dehydrated in a series of graded ethanol, cleared in xylene, and mounted in DPX (BDH/Merck, Leicestershire, UK). The negative control slides consisted of a section of the tissue block being studied, which was treated identically to all other slides, with the exception that the primary antibody was omitted to control for nonspecific binding of the detection system.

For evaluation and scoring of immunohistochemical staining, the sections were observed on a Labor Lux microscope (Leitz, Wetzlar, Germany), at a magnification of ×100, ×200, and ×400. A positive reaction was characterized by the presence of brown staining. Each section was examined by two observers who were blinded to the source of tissue and the intensity of staining was assessed using “*H* score” which was calculated using the following equation [25]: *H* score = Σ*P*_*ί*_ (*ί* + 1), where *ί* represents the intensity of staining (0 = negative; 1 = weak; 2 = moderate; and 3 = strong) and *P*_*ί*_ is the percentage of cells (0–100%) stained at each intensity. In the case of a wide disagreement between the two observers, the slides were reanalyzed by a third independent reviewer. The final result was obtained by averaging the individual observer results. Representative sections were photographed using an Olympus digital camera at ×100 magnification.

### 2.5. Measurement of Extracted Protein Concentrations

The concentrations of the total proteins extracted from the liver specimens were measured using the BioSpec-nano (Shimadzu Corporation, Japan) at 280 OD. All protein samples were diluted using normal saline to make a final concentration of 500 *μ*g/mL as previously described [26].

### 2.6. Enzyme Linked Immunosorbent Assay (ELISA)

ELISA was used for quantitative measurement of serum and liver activin-A and follistatin (R&D systems, Minneapolis, USA). All samples were processed in duplicate and according to the manufacturer's instructions. The optical density of the plates was measured within 10 min using a plate reader at 450 nm and correction at 560 nm as recommended by the manufacturer.

As reported by the manufacturer, the lowest detection limit of activin-A by the used kit is 3.7 pg/mL and the upper limit is 1500 pg/mL. The intra-assay and interassay precisions of the kit are 4.3% and 5.8%, respectively. The kit cross reacts by 0.2% and 0.45% with inhibin-A and activin-AB, respectively. The detection range of the follistatin kit is 250–16000 pg/mL and the minimum detectable dose is 83 pg/mL.

### 2.7. RNA Extraction and cDNA Synthesis

Total RNA was isolated from the stored liver specimens in RNA*later* following homogenisation of the specimens and by using the Purelink RNA mini kit from Life Technologies (Thermo Fisher Scientific, CA, USA) and according to the manufacturer's instructions. RNA was treated with RNAse-free DNAse during the extraction protocol to avoid the collection of genomic DNA and the concentrations and quality of the extracted total RNA were measured using the BioSpec-nano (Shimadzu Corporation, Japan), and its quality and integrity were concluded through the A260/A280 ratio.

For cDNA synthesis, 200 ng of total RNA was transcribed to cDNA using a high capacity RNA-to-cDNA Reverse Transcription Kit from Applied Biosystems (Thermo Fisher Scientific, Warrington, UK), following the manufacturer's protocol.

### 2.8. Quantitative RT-PCR

Quantitative RT-PCR was performed using the 2^−ΔΔCt^ method on the following 6 target rat genes: activin *β*A-subunit (NM_017128.2), activin type IIA receptor (NM_031571.2), activin type IIB receptor (NM_031554.1), follistatin (NM_012561.1), Smad4 (NM_019275.2), and Smad7 (NM_030858.1). The results were normalised against the Ct values of *β*-actin (NM_031144.3) and expressed as fold-change compared with the normal control group. The nucleotides primer sequences of these 7 rat origin genes are summarized in Table 1.

PCR reactions were carried out by using power SYBR Green master mix from Applied Biosystems (Thermo Fisher Scientific, Warrington, UK) and a step one Real Time PCR system (Applied Biosystems, USA) in triplicate wells. Each well of the PCR plate contained 10 *μ*L SYBR Green, 7 *μ*L DNase/RNase-free water, 1 *μ*L of each primer (5 pmol), and 1 *μ*L cDNA (25 ng). The amplification was performed under the following conditions: 40 cycles (95°C 15 s and 60°C 1 min). Two negative controls were included, one with minus-reverse transcription (minus-RT) control from the previous reverse transcription step and a minus-template PCR, which contained all the PCR components, but water was used as a template.

### 2.9. Statistical Analysis

Statistical analysis of the results was performed using SPSS version 16. Normality and homogeneity of data were assessed with the Kolmogorov and Smirnoff test and Levene test, respectively. One-way ANOVA followed by Tukey's post hoc test or Kruskal-Wallis followed by Dunn's post hoc test was used to compare between the different groups depending on the data homogeneity. Correlations were determined using Pearson's test. *P* value <0.05 was considered significant.

## 3. Results

### 3.1. Results of Routine Biochemistry

There was no significant difference (*P* > 0.05) using one way ANOVA between the different study groups in body weight, liver weight, liver enzymes, metabolic profile, and renal function parameters (Table 2).

### 3.2. Immunohistochemistry

**Figure 1:**
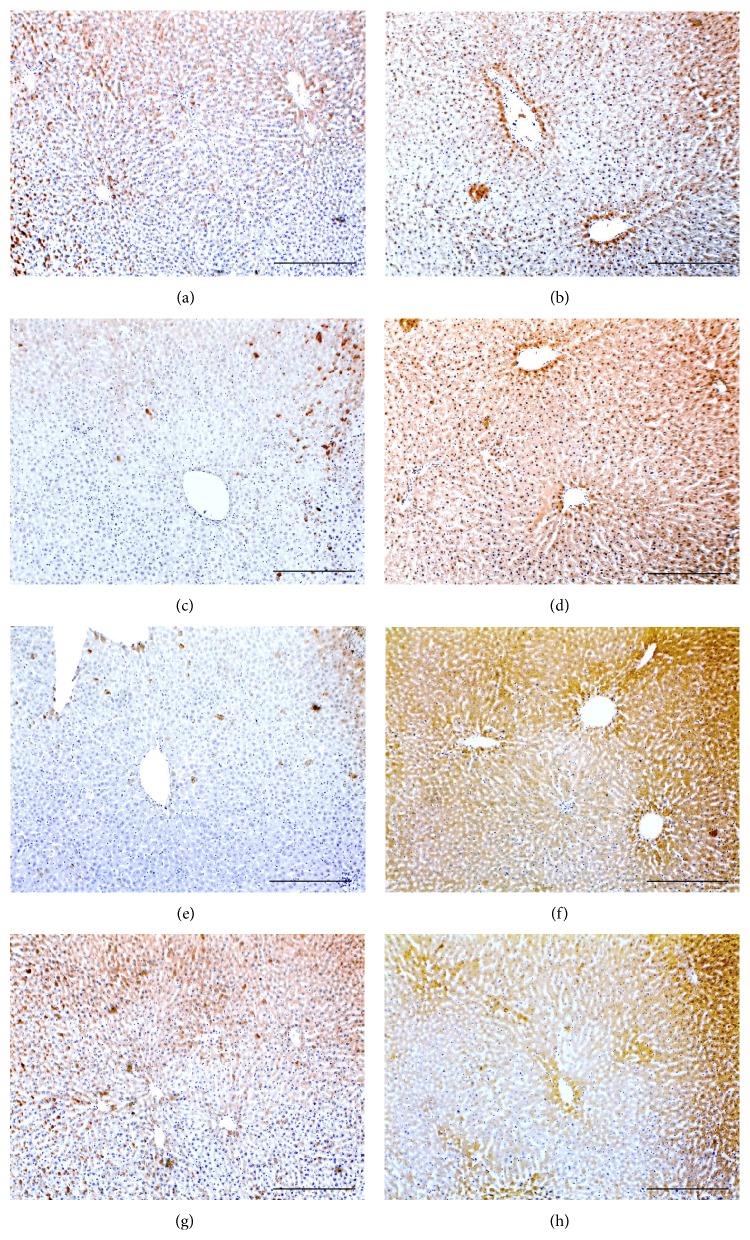
Immunohistochemistry localisation of activin *β*A-subunit (left column) and follistatin (right column) in control (a and b), Peg-only (c and d), Peg & RBV (e and f), and RBV-only (g and h) groups (×100 magnification, scale bar = 15 *μ*m).

**Figure 2:**
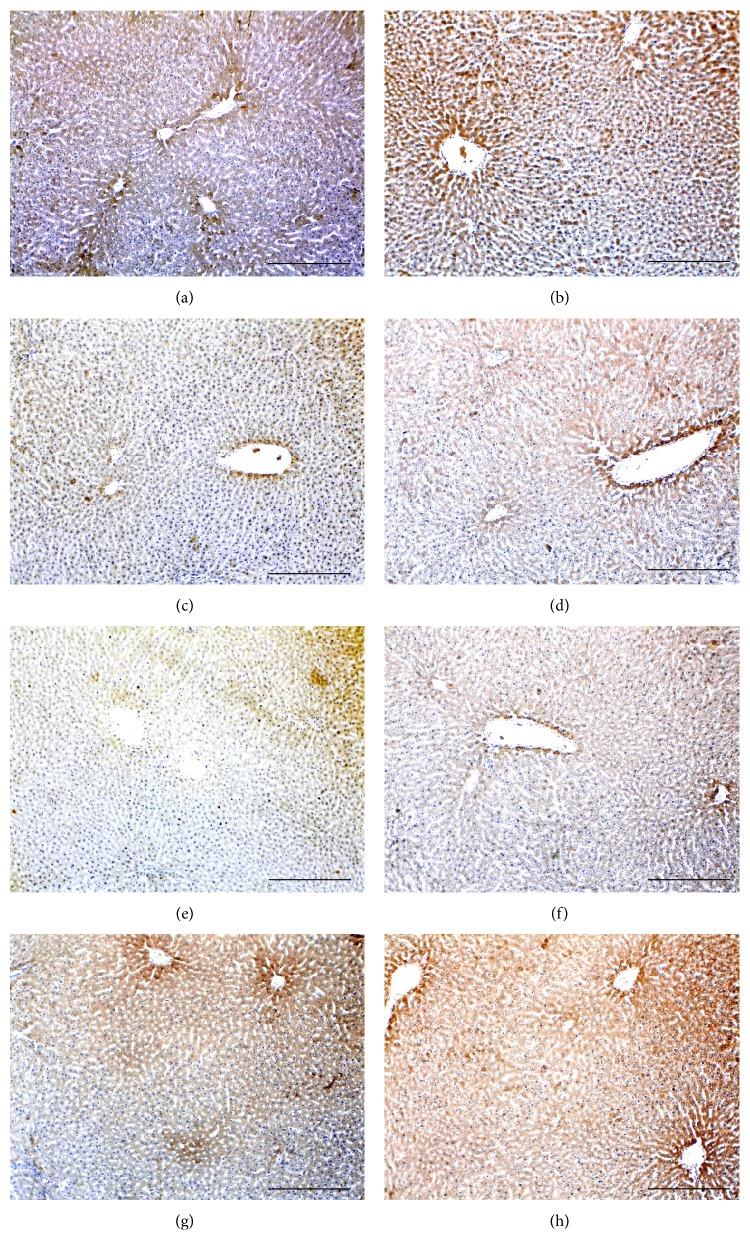
Immunohistochemistry localisation of activin type IIA (left column) and IIB (right column) receptors in control (a and b), Peg-only (c and d), Peg & RBV (e and f), and RBV-only (g and h) groups (×100 magnification, scale bar = 15 *μ*m).

**Figure 3:**
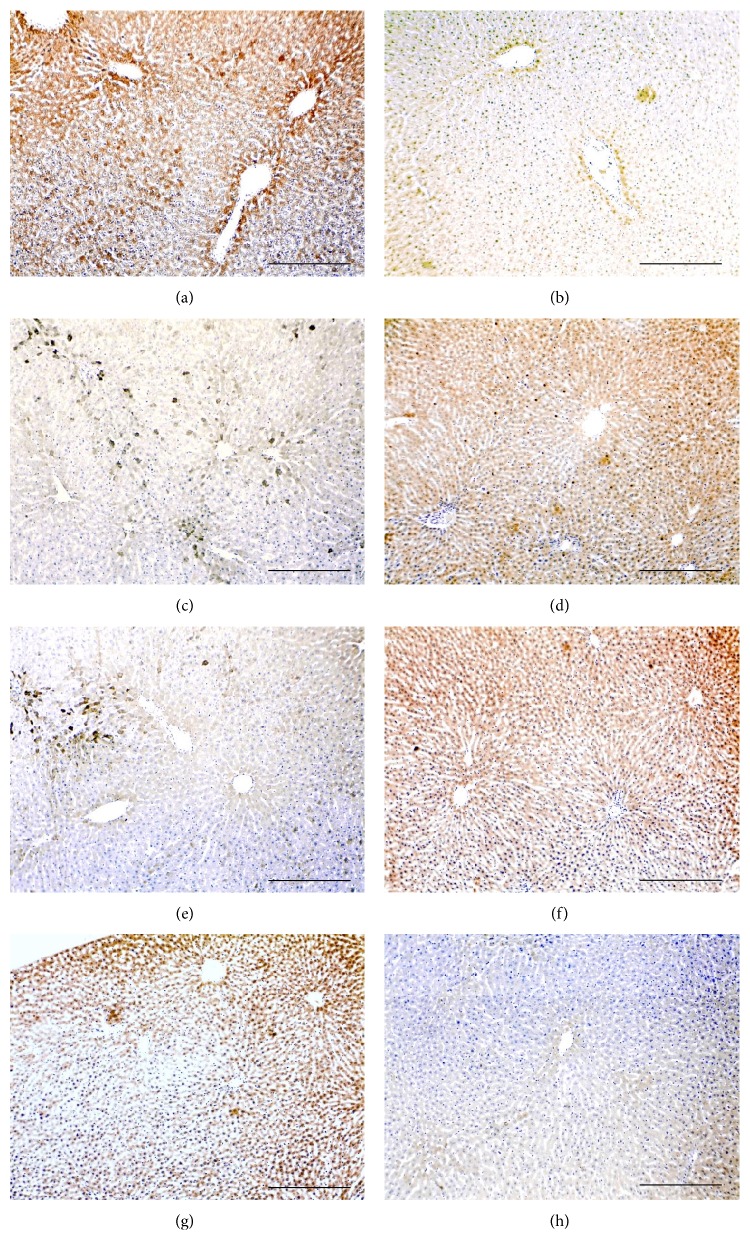
Immunohistochemistry localisation of Smad4 (left column) and Smad7 (right column) in control (a and b), Peg-only (c and d), Peg & RBV (e and f), and RBV-only (g and h) groups (×100 magnification, scale bar = 15 *μ*m).

All molecules were detected in the liver sections of all groups and the immunostain was localised in the cell membrane and cytoplasm of hepatocyte surrounding hepatic vessels (Figures 1–3). Furthermore, Smad4 and Smad7 exhibited nuclear localisation in the stained hepatocytes.

The use of Peg-INF-*α*-2a either alone (Figures 1(c) and 1(d)) or in combination with RBV (Figures 1(e) and 1(f)) significantly decreased the expression of activin *β*A-subunit (Figures 1(c) and 1(e)) and significantly increased the expression of follistatin (Figures 1(d) and 1(f)) compared with “control” (Figures 1(a) and 1(b)) and “RBV-only” groups (Figures 1(g) and 1(h)). Similar to activin, the expression of type IIA and IIB receptors (Figure 2) and Smad4 (Figures 3(c)–3(f)) was also significantly decreased in the “Peg-INF-only” group (Figures 2 and 3(c) and 3(d)) and “Peg-INF and RBV” group (Figures 2 and 3(e) and 3(f)) compared with the other groups (Table 3).

Smad7 showed a similar pattern of expression to follistatin as it was significantly higher in the group injected with Peg-INF-*α*-2a alone (Figure 3(d)) or in combination with RBV (Figure 3(f)) compared with control (Figure 3(a)) and RBV-only (Figure 3(h)) groups. There was no significant difference in the expression of the candidate molecules between “RBV-only” and “control” groups (*P* > 0.05).

### 3.3. Concentrations of Serum and Liver Activin-A and Follistatin

Administration of Peg-INF-*α*-2a for 4 weeks significantly decreased the concentrations of activin-A and significantly increased the concentrations of follistatin at both serum and liver levels of the “Peg-only” and “Peg & RBV” groups compared to “control” group. Furthermore, significant differences were detected between “Peg & RBV” and “Peg-only” in serum, but not liver, concentrations of candidate proteins (Table 4).

There was no significant difference in the concentrations of candidate proteins in the “RBV-only” and “control” groups at the serum and liver levels. However, a significant difference was detected in “RBV-only” compared to “Peg-only” and “Peg & RBV” groups in serum and liver concentrations of activin-A and follistatin.

### 3.4. Correlations between Serum and Liver Activin-A and Follistatin

**Figure 4:**
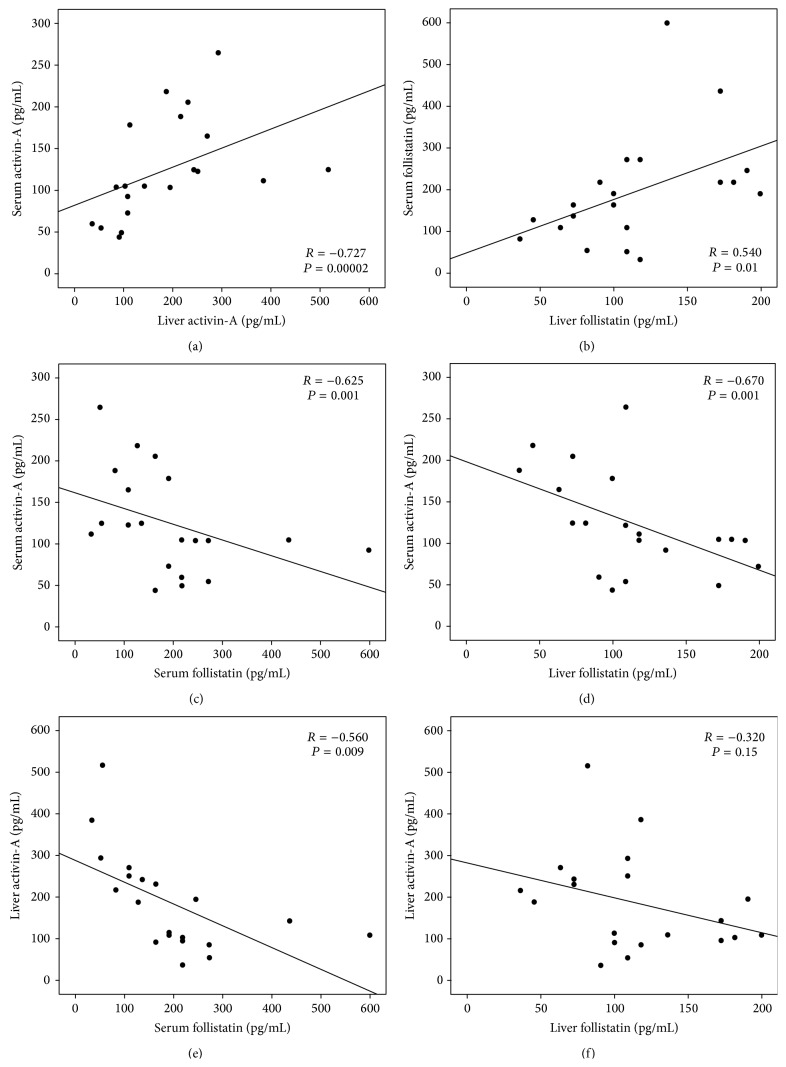
Correlation between (a) serum and liver activin-A, (b) serum and liver follistatin, (c) serum activin-A and serum follistatin, (d) serum activin-A and liver follistatin, (e) liver activin-A and serum follistatin, and (f) liver activin-A and follistatin by Pearson's correlation test.

There was a significant positive correlation between serum and liver concentrations of activin-A (*r* = 0.727, *P* = 0.02 × 10^−3^) and follistatin (*r* = 0.540, *P* = 0.01). Serum activin-A also correlated negatively and significantly with serum follistatin (*r* = −0.625, *P* = 0.001) and liver follistatin (*r* = −0.674, *P* = 0.001). Additionally, a significant negative correlation was seen between liver activin-A and serum follistatin (*r* = −0.560, *P* = 0.009). However, there was a nonsignificant correlation between liver activin-A and liver follistatin (Figure 4).

### 3.5. Quantitative RT-PCR

**Figure 5:**
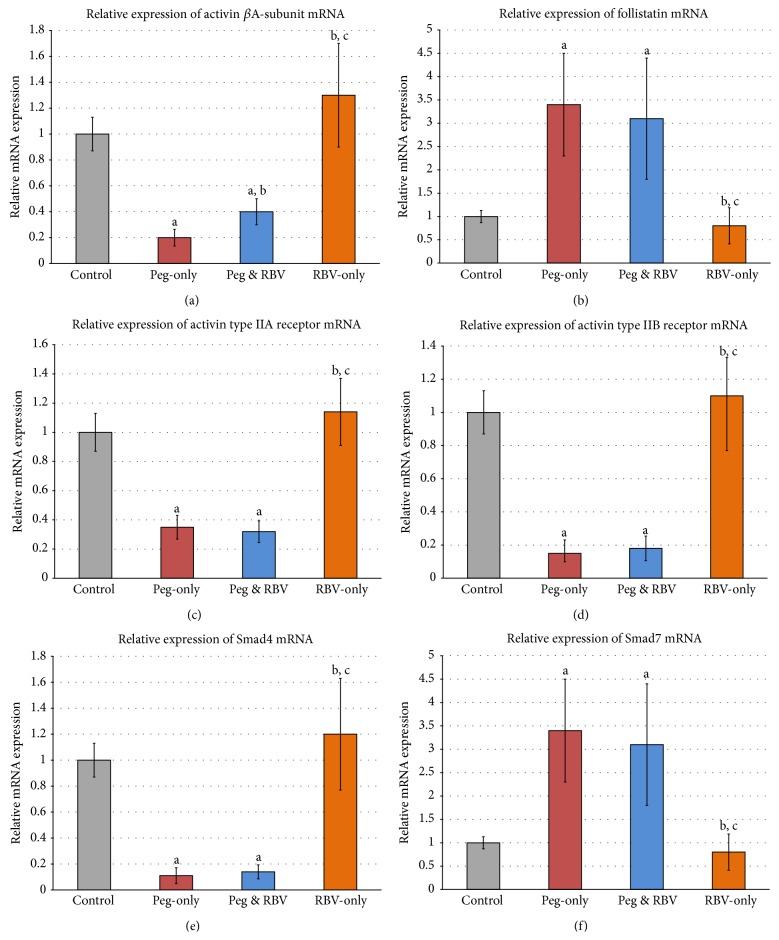
Relative concentration of messenger RNA expression of (a) activin *β*A-subunit, (b) follistatin, (c) type IIA receptor, (d) type IIB receptor, (e) Smad4, and (f) Smad7 in the different study groups (^a^ *P* < 0.05 compared to control, ^b^ *P* < 0.05 compared to Peg-only, and ^c^ *P* < 0.05 compared to Peg & RBV group).

Gene expression study showed a significant decrease in the mRNA expression of activin *β*A-subunit (5-fold), type IIA (3.8-fold) and type IIB (5-fold) receptors, and Smad4 (7-fold) in the groups injected with Peg-INF-*α*-2a (Figure 5). Furthermore, a significant increase was observed (*P* < 0.05) in the gene expression of follistatin (3-fold) and Smad7 (3-fold) in the “Peg-INF-*α*” and “Peg-INF-*α* and RBV” groups when compared with the “control” and “RBV” groups (Figure 5). Nevertheless, there was no significant difference in the expression of the genes of interest between “RBV” and “control” groups (*P* > 0.05).

## 4. Discussion

To the best of our knowledge, this is the first study to report the effect of Peg-INF-*α* and ribavirin on the expression of activin-A, its type II receptors, its intracellular mediators, and follistatin in liver specimens collected from experimental animal model. Our results demonstrated at the gene and protein levels that Peg-INF-*α* significantly decreased the expression of activin *β*A-subunit, activin type IIA and IIB receptors, and Smad4 in “Peg-only” and “Peg & RBV” groups compared with “control” and “RBV-only” groups. Simultaneously, the injection of the drug for 4 weeks was also associated with a significant increase in the expression of follistatin and Smad7 mRNAs and proteins by the hepatocyte. Furthermore, similar observations were also noted for the serum concentrations of mature activin-A and follistatin proteins which correlated significantly with the concentrations of activin-A and follistatin in the liver.

Our results suggest that activin-A and its related proteins are potential targets for interferon-*α* and Peg-INF-*α*, but not ribavirin, modulates the production and actions of these proteins in the liver. Furthermore, the variations in the liver concentrations of activin-A and follistatin mature proteins following Peg-INF-*α* are reflected and could be detected in serum.

Activins are dimer proteins that consist of two *β*-subunits (*β*A- and *β*B-subunits) and the different dimerization of subunits gives rise to three proteins: activin-A (*β*A-*β*A), activin-B (*β*B-*β*B), and activin-AB (*β*A-*β*B) [3]. Activins initiate their actions by binding to their cell surface type IIA and IIB receptors, leading to the recruitment of type I receptor followed by a cascade of phosphorylation reactions of their intracellular mediators known as Smads to propagate the signal to the nucleus [15]. Activins and their related molecules are expressed in the liver of several species and they are mainly involved in the regulation of hepatocyte regeneration and differentiation [27, 28]. Several studies have shown that activin-A induces hepatocyte growth arrest and apoptosis in vivo and in vitro and these actions are Smad dependent [29–32]. These effects can be blocked by the activin binding protein, follistatin, which has been demonstrated to promote the growth and differentiation of liver cells [33–35]. Hence, it has been suggested that activins are tightly regulated in the liver and pathological alterations in the activin-follistatin axis have been associated with several hepatic disorders including viral and nonviral diseases [20, 21, 36–39].

Our results correlate with the previous studies as they showed the expression of activin *β*A-subunit, activin type IIA and IIB receptors, its binding protein, and its intracellular mediators Smad4 and Smad7. Our results support the assumption that activins act through paracrine/autocrine mode of action in the liver and their functions are tightly regulated in the liver.

Activins and follistatin are also involved in the regulation of the immune system and pathological expression of these proteins has been reported in a variety of immune and inflammatory responses [40–42]. Furthermore, the production and expression of activins, their intracellular mediators, and follistatin have been shown to be modulated by interferons in a variety of tissues. Treatment of patients with hepatitis B virus with IFN-*γ* resulted in a decrease in the fibrosis scores and it has been suggested that the antifibrotic effects of the drug were achieved by upregulating Smad7 expression and impairing the signalling of TGF-*β* through inhibition of Smad4 [43]. Additionally, treating keloid derived fibroblasts with INF-*γ* significantly increased the expression of Smad3 and Smad7 in a time and dose dependent manner [44]. The use of recombinant human interferon-*β*1a in vitro on hepatic stellate cells has also shown dose dependent antifibrotic activities by decreasing the expression of Smad4 and collagen type I and collagen type III with a concurrent increase in the expression of the inhibitory Smad7 [45].

Interferon-*γ* has also been shown to suppress the actions of activin-A by reciprocally regulating the secretion of activin-A and follistatin from bone marrow stromal fibroblasts [46]. A more recent study has also reported that INF-*γ* regulates the host immune cells and inhibits alternative macrophage activation during allograft rejection by decreasing the expression of activin *β*A- and *β*B-subunits [47]. Similar suppressive effects have also been recently reported for INF-*γ* on the activities of activin-A during globin gene expression [48]. We have previously reported that the serum concentrations of activin-A increase dramatically in patients with CHC and they correlated significantly with the serum levels of IL-6, TNF-*α*, and the severity of liver damage associated with CHC [22]. Later, we have also demonstrated that serum activin-A and follistatin were modulated during the treatment of CHC with Peg-INF-*α* based therapy and that their levels returned to normal in the responder group [23].

The current findings are in agreement with the previous studies as we observed a significant decrease in the expression of activin *β*A-subunit, activin type II receptors, and Smad4 in the liver following the use of Peg-INF-*α* for 4 weeks, which synchronized with a significant increase in the expression of follistatin and inhibitory Smad7. Our results suggest that Peg-INF-*α* inhibits the actions of activin-A in the liver by increasing its binding protein and inhibitory Smad7, downregulating activin *β*A-subunit, and inhibiting the production of activin type II receptors and Smad4.

Hence, we hypothesise that activins and their related proteins are potential targets for Peg-INF-*α* based therapy during the treatment of CHC. In this regard, activin-A has been shown to induce deviation of immune response towards a type 2 phenotype [49], and Peg-INF-*α* eradicates the viral infection by altering the immune response in patients with CHC from Th2 to a Th1 mediated pattern [8]. Peg-INF-*α* based therapy also increases the expression of toll-like receptors 2 and 4 in patients with CHC [50], which are potent regulators of the release of activin-A [51, 52]. Additionally, the production of TNF-*α* in the natural killer cells increases and that of serum IL-6 and serum IL-10 decreases following Peg-INF-*α* [13, 53]. These cytokines have also been shown to be regulated by activin-A [54]. Moreover, the release of INF-*γ*, which plays an important role in controlling CHC following Peg-INF-*α* based therapy [55], is regulated by activin-A [47].

Our findings of a significant decrease in dimeric activin-A and significant increase in follistatin at the serum and liver levels following the administration of Peg-INF-*α* compared to “control” and “RBV” groups further support our hypothesis that Peg-INF-*α* alters the production of these proteins at the liver and serum levels. Additionally, the observed significant positive correlations between liver and serum concentrations of activin-A and follistatin suggest that the liver is a major source of these proteins in serum and that alteration in the hepatic volume of these proteins following injection of Peg-INF-*α* is reflected and detected at the serum level. Further studies are required to illustrate the mechanism(s) by which Peg-INF-*α* regulate the production of activin-A and follistatin by the liver.

Although follistatin is regarded as the activin binding protein [56], it appears that the production of follistatin is not only driven by activin during inflammation [57, 58]. The synthesis and secretion of follistatin are also modulated by other cytokines, including IL-1*β*, TNF-*α*, and IFN-*γ* [46, 59]. The physiological and pathological activities of activin-A are usually antagonised by follistatin at the cellular and serum levels [56]. Our results agree with these findings as they have shown a significant increase in hepatic and serum concentrations of follistatin following treatment with Peg-INF-*α*, suggesting that the drug controls the activities of activin-A by decreasing its production and increasing its binding protein in liver and serum. Additionally, the observed alterations in the expression of activin type II receptors and activin intracellular mediators suggest that Peg-INF-*α* could regulate the actions of activins in the liver by inhibiting the propagation of the signal through multipathway mechanisms.

Ribavirin monotherapy is not effective in the treatment of CHC and a number of studies have suggested that strong antiviral activity is only seen when RBV is combined with either INF-*α* or Peg-INF-*α*. Therefore, the presence of synergism between Peg-INF-*α* and RBV has been suggested, which has been shown in vitro [60]. Additionally, it has been suggested that RBV may have immune modulatory activities, including the regulation of macrophage and T helper cells produced cytokines, modulation of the Th1/Th2 subset balance, and the enhancement of the expression of interferon stimulated genes [61].

Our results support the previous findings as they have demonstrated a significant decrease in serum activin-A and significant increase in serum follistatin in “Peg & RBV” group compared to “Peg-only” and “RBV-only” groups. However, these synergistic alterations were only detected at the serum level, suggesting that combined effect of Peg-INF-*α* with ribavirin modulates the production of the candidate molecules at other organs/systems beside the liver, affecting the concentrations of these proteins at the serum level.

The biological activities of activins are tightly regulated by follistatin as the binding of activin to follistatin is almost irreversible [15]. Serum activin is commonly bound with the long form of follistatin (FS-315) [62], while the short form of (FS-288) has high affinity for cell membrane activins [63]. The currently available ELISA kits for the detection of activin-A and follistatin cannot distinguish between the free and bound forms of both proteins. Furthermore, the follistatin kit measures both the long and short forms. Therefore, the reported results in our study are shown at the level of total activin-A and follistatin and the development of ELISA kits that measure the free form of both proteins would expose precisely the effects of Peg-INF-*α* on the activity of both proteins.

In conclusion, Peg-INF-*α* modulates the production of activin-A, its type IIA and IIB receptors, intracellular mediators, and binding protein by the liver, which appears to be a major source of activin-A and follistatin. Alterations in the concentrations of activin-A and follistatin in the liver are reflected and can be detected at the serum level. Further studies are needed to explore the role of Peg-INF-*α* based therapy on the production of activins and follistatin by the liver and immune cells.

## Figures and Tables

**Table 1:** The sequences of PCR primers used for the detection of *β*-actin, activin *β*A-subunit, activin type IIA and IIB receptors, Smad4, Smad7, and follistatin including the corresponding genes accession numbers.

	Forward	Reverse
*β*-actin (NCBI: NM_031144.3)	5′ CGG TCA GGT CAT CAC TAT CG 3′	5′ TTC CAT ACC CAG GAA GGA AG 3′
*β*A-subunit (NCBI: NM_017128.2)	5′ GCT TTG GCT GAG AGG ATT TCT G 3′	5′ TGG ATT ATA GTG AGG AGT TCC 3′
IIA receptor (NCBI: NM_031571.2)	5′ AGG CTA ATG TGG TCT CTT GGA A 3′	5′ CCA ATC CTC TAG CCA TGG TTT CT 3′
IIB receptor (NCBI: NM_031554.1)	5′ GGA GTG CAT CTA CTA CAA CGC 3′	5′ TCC AGG CCG CTC TGG TT 3′
Smad4 (NCBI: NM_019275.2)	5′ GTG GCT GGT CGG AAA GGA TTT 3′	5′ GAT CAG GCC ACC TCC AGA GAC 3′
Smad7 (NCBI: NM_030858.1)	5′ GGA GGT CAT GTT CGC TCC TT 3′	5′ TTT GGT CCT GAA CAT GCG GG 3′
Follistatin (NCBI: NM_012561.1)	5′ GTG GAT GAT TTT CAA CGG GGG 3′	5′ TTC TCA CAC GTT TCT TTA CAG GG 3′

**Table 2:** Mean ± SD of body weight, total liver weight, liver enzymes, albumin, renal function, and metabolic parameters in the different study groups.

	Control	Peg-INF only	Peg-INF & ribavirin	Ribavirin only
Body weight (g)	221.57 ± 20.01	231.97 ± 23.01	218.42 ± 13.64	230.1 ± 22.2
Liver weight (g)	11.04 ± 1.26	10.54 ± 1.52	10.27 ± 0.44	10.23 ± 1.5
ALT (U/L)	60.4 ± 16.2	68.8 ± 18.6	53.9 ± 19	64.1 ± 11.5
AST (U/L)	92.4 ± 24.2	129.8 ± 46.7	119 ± 21.6	133 ± 49.8
Albumin (g/dL)	3.4 ± 0.4	3.3 ± 0.5	3.7 ± 0.3	3.5 ± 0.5
Creatinine (mg/dL)	0.22 ± 0.03	0.2 ± 0.06	0.2 ± 0.03	0.19 ± 0.03
Urea (mg/dL)	47.6 ± 5.1	52.3 ± 4	56.6 ± 9.5	47.3 ± 5.8
BUN (mg/dL)	22.2 ± 2.4	24.4 ± 1.9	26.3 ± 4.4	22 ± 2.7
Glucose (mg/dL)	110.7 ± 12.7	118.9 ± 23.1	112.6 ± 12.7	114.5 ± 16.1
Triglycerides (mg/dL)	77.3 ± 20.3	68.3 ± 13.9	63.2 ± 9.5	85 ± 28.5
Cholesterol (mg/dL)	45 ± 7.2	58.5 ± 12.4	62.2 ± 14.5	64.8 ± 9.7
HDL-C (g/dL)	36.2 ± 9.2	44.3 ± 1.2	52.8 ± 7.8	53.1 ± 7.7
LDL-C (g/dL)	6.6 ± 1.7	8.14 ± 2.33	7.1 ± 2.5	6.3 ± 1.55

**Table 3:** Mean ± SD of immunohistochemistry scores for activin *β*A-subunit, activin type receptor IIA and IIB, Smad4, Smad7, and follistatin proteins in liver specimens.

	Normal group	Peg-only group	Peg & RBV group	RBV-only group
*β*A-subunit	215.5 ± 31.2	96.4 ± 23.4^a^	111.48 ± 44.3^a^	223.9 ± 38.5^b,c^
Receptor IIA	283.7 ± 58.7	121.4 ± 29.2^a^	135.7 ± 27.6^a^	269.2 ± 46.8^a,b^
Receptor IIB	332.7 ± 67.6	176.5 ± 32.6^a^	202.4 ± 41.8^a,b^	294.7 ± 38.6^b,c^
Smad4	301.1 ± 26	47.2 ± 20.3^a^	66.1 ± 23.9^a^	258.7 ± 31.6^a,b,c^
Smad7	61 ± 26.7	270.6 ± 68.7^a^	267.6 ± 55.3^a^	89.6 ± 30.1^b,c^
Follistatin	159.4 ± 9.5	373.1 ± 66.8^a^	351.8 ± 47.2^a^	178.1 ± 43.6^b,c^

^a^ *P* < 0.05 compared with normal; ^b^ *P* < 0.05 compared with Peg-only group; and ^c^ *P* < 0.05 compared with Peg & RBV group.

**Table 4:** Mean ± SD of serum and liver concentrations of activin-A and follistatin in the different groups.

	Activin-A (pg/mL)		Follistatin (pg/mL)	
	Serum	Liver	Serum	Liver
Control	166.5 ± 41.7	216.1 ± 45.7	128.9 ± 40.9	72.6 ± 22.1
Peg-only	92.08 ± 15.3^a^	115.2 ± 37.5^a^	354.15 ± 91.1^a^	157.4 ± 28.5^a^
Peg & RBV	56.5 ± 10.9^a,b^	112.4 ± 41.6^a^	212.4 ± 40.4^a,b^	134.4 ± 38.6^a^
RBV-only	174.7 ± 35.8^b,c^	281.8 ± 84.2^b,c^	98.9 ± 33.4^b,c^	82.2 ± 23.5^b,c^

^a^ *P* < 0.05 compared to control, ^b^ *P* < 0.05 compared to Peg-only, and ^c^ *P* < 0.05 compared to Peg & RBV group.
